# Homologous recombination deficiency (HRD) score in aggressive prostatic adenocarcinoma with or without intraductal carcinoma of the prostate (IDC-P)

**DOI:** 10.1186/s12916-022-02430-0

**Published:** 2022-07-22

**Authors:** Sha Zhu, Jinge Zhao, Ling Nie, Wenlian Yin, Yaowen Zhang, Fengnian Zhao, Yuchao Ni, Xingming Zhang, Zhipeng Wang, Jindong Dai, Zhenhua Liu, Junru Chen, Yuhao Zeng, Zilin Wang, Guangxi Sun, Jiayu Liang, Xiaochen Zhao, Xudong Zhu, Ronggui Tao, Jiyu Yang, Ben He, Ni Chen, Pengfei Shen, Hao Zeng

**Affiliations:** 1 Department of Urology, Institute of Urology, West China Hospital, Sichuan University, Chengdu, 610041 China; 2 Department of Pathology, West China Hospital, Sichuan University, Chengdu, 610041 China; 3 3D Medicines Inc., Shanghai, China

**Keywords:** Homologous recombination deficiency, Genomic instability, HRD score, Prostate cancer, IDC-P

## Abstract

**Background:**

Intraductal carcinoma of the prostate (IDC-P) is a subtype of prostate cancer featured by poor prognosis. Previous studies suggested IDC-P could have a potentially unstable genome. Homologous recombination deficiency (HRD) score is a result-oriented method to describe the genomic instability status. This study investigates the association of HRD scores with IDC-P and other clinicopathological factors and the prognostic implication of HRD scores in an aggressive prostate cancer cohort.

**Methods:**

This study involved 123 PCa patients, including high-risk localized (M0) and de novo metastatic (M1) diseases. HRD score is calculated based on over 10,000 single-nucleotide polymorphisms distributed across the human genome. We explored the association between HRD scores and clinicopathological characteristics, genomic alterations, and patients’ prognoses using rank-sum tests, chi-square tests, Kaplan-Meier curves, and Cox proportional hazards method.

**Results:**

The median HRD score of this cohort is 21.0, with 65 (52.8%) patients showing HRD score≥21. Tumors with IDC-P displayed higher HRD scores than adenocarcinoma (*P*=0.002); other high HRD score-related factors included M1 (*P* =0.008) and high ISUP grades (4–5) (*P*=0.001). *MYC* mutations were associated with high HRD scores (*P*<0.001) in the total cohort. *TP53* mutations (*P*=0.010) and HRR pathway mutations (*P*=0.028) corresponded to high HRD scores in IDC-P positive and non-IDC-P patients, respectively, but not vice versa. HRD scores higher than 21 indicated significantly worse survival in the total cohort.

**Conclusions:**

M1, high Gleason score, and IDC-P pathology represent higher HRD scores in PCa. Tumors with IDC-P might have different driven mechanisms for high HRD scores than non-IDC-P. HRD score displayed prognostic value in this aggressive prostate cancer cohort.

**Supplementary Information:**

The online version contains supplementary material available at 10.1186/s12916-022-02430-0.

## Background

Genome instability is a hallmark of cancer. Homologous recombination deficiency (HRD) contributes significantly to the unstable genome and can be targeted by poly ADP-ribose polymerase inhibitors (PARPis). The therapeutic effect of PARPis has been acknowledged in ovarian, breast, pancreatic, and prostate cancer (PCa) [1]. However, current detecting methods are inadequate to identify all PARPi-sensitive patients.

HRD is a bonafide treatment target, but questions remain in defining HRD status accurately. Around 20% PCa patients show DNA damage repair (DDR) gene defects, and they are likely to harbor more aggressive diseases [2–4]. Although germline DDR, especially *BRCA* mutations, remain the acknowledged clinical predictor for PARPi usage, DDR mutations are insufficient to describe the whole picture of patients’ HRD status. Furthermore, genomic testing on DDR mutations suffers from a few inherent caveats: reversion mutation, epigenetic regulation, and potential predictive mutation from other genes, especially in PCa where biallelic gene loss exclusively by somatic events is prominent [5]. Therefore, describing the HRD state from a broader view is being constantly explored. Recent years have seen the emergence of multiple tests for HRD status, including mutational, genomic, transcriptional, and cytological signatures. With the mutational signature-based algorithm (HRDetect) (to detect *BRCA1/BRCA2*-deficient tumors) having identified HRD tumors without homologous recombination repair (HRR) mutations, we have reasons to hypothesize alternative factors in contributing to HRD [6].

HRD score is a test based on the measurement of chromosomal aberrations, a consequent reflection of a defective upstream DNA double-strand break precise repair. The calculation of HRD score derives from the incorporation of three SNP-based assays: loss of heterozygosity (LOH), telomeric allelic imbalance (TAI), and large-scale state transition (LST), corresponding to three discrete types of gross genomic aberration [7]. Tumor’s HRD score directly shows the consequence of the loss of HRR function (genomic scars), irrespective of which part of the pathway went wrong. Thus, the HRD score may be a more reliable biomarker for identifying potential sensitive patients for PARPi than mutations in HRR-related genes.

However, the detailed HRD score distribution in PCa is yet to be depicted. Current pan-studies show contradictory results regarding PCa [5, 8]. A deeper understanding of the HRD score in PCa will allow better patient stratification to HRD-targeted therapies. In addition, PCa is marked by its heterogeneity regarding pathological subtypes, while these aspects seem to be understudied. Our team has reported the prognostic and molecular discrepancy among different PCa pathological subtypes [9–12], particularly interested in the intraductal carcinoma of the prostate (IDC-P) subtype. Of note, apart from its association with negative clinicopathological characteristics and poor prognosis, IDC-P is different from acinar adenocarcinoma (AC) in terms of genomic and transcriptomic features [13]. Previous studies have shown that IDC-P harbors a higher percentage of genomic aberrations in IDC-P [14, 15]. Hence, we hypothesize that tumors with IDC-P components harbor increased genomic scars, thus manifesting high HRD scores.

This study investigates the association of HRD scores with IDC-P and the prognostic value of HRD scores in an aggressive PCa cohort containing high-risk localized (M0) and de novo metastatic (M1) PCa patients. We also explore the possible correlations of HRD scores with genomic aberrations other than HRR pathway.

## Patients and methods

### Patients

Protocols in this study obtained West China Hospital institutional review board approval. We started by screening all prostate biopsies and prostatectomy samples with detailed pathological reports in our institute within 5 years. These samples were all pathologically re-evaluated, and samples with >20% tumor content were considered for subsequent inclusion. We contacted these patients with qualified samples; those who agreed to participate and were able to provide written consent and blood samples were included in this study.

Finally, this study included 123 patients diagnosed with PCa between 2015 and 2020 in West China Hospital, including 46/123 (37.4%) M0 PCa and 77/123 (62.6%) M1 PCa patients. HRD score and genomic alteration testing were performed using the tissue from prostate biopsies or radical prostatectomy (RP) specimens at first diagnosis.

### Clinical data collection, pathological review, and IDC-P diagnosis

We collected the included patients’ age, baseline prostate-specific antigen (PSA), metastasis status and sites, pathological reports, and treatment history. Two independent pathologists (Ni Chen and Ling Nie) performed pathological reviews to reconfirm IDC-P for each sample.

IDC-P diagnosis for each patient was made based on morphological characteristics and three immunohistochemistry markers (HCK, p63, and AMACR), in which the HCK and P63 could clearly stain the basal cells. IDC-P subtypes were reported according to the criteria in the 2016 WHO classification. IDC-P subtype classification was based on our previous report: pattern 1—loose cribriform or micropapillary pattern with either marked nuclear atypia or comedonecrosis, and pattern 2—solid or dense cribriform pattern [9].

### Tissue collection, processing, and genomic DNA extraction

Genomic DNA was extracted from diagnostic formalin-fixed paraffin-embedded (FFPE) tissue sections evaluating tumor cell content using hematoxylin and eosin (H&E) staining, containing only ≥ 20% tumor cells for subsequent analyses. Total DNA was isolated from 5-μm FFPE sections which were placed in a 1.5-ml microcentrifuge tube and dissolved the paraffin using mineral oil, followed by lysis buffer and proteinase K at 56 °C overnight until the tissue was completely digested. Subsequently, the resulting cell lysate was incubated at 80 °C for 4 h to reverse the protein-DNA formaldehyde crosslinks. Genomic DNA was extracted from tissue samples using the ReliaPrep™ FFPE gDNA Miniprep System (Promega) and quantified using the Qubit™ dsDNA HS Assay Kit (Thermo Fisher Scientific) following the manufacturer’s protocol.

### Library preparation and targeted capture

DNA extracts (30–200 ng) were sheared to an average fragment size of 250 bp using an S220 focused-ultrasonicator (Covaris). According to the manufacturer’s protocol, libraries were prepared using the KAPA Hyper Prep Kit (KAPA Biosystems). Each library’s fragment size and concentration distribution were assessed on a LabChip GX Touch HT Analyzer (PerkinElmer) and a Qubit 3.0 fluorometer (Thermo Fisher Scientific), respectively.

Targeted capture was performed by probe-based hybridization with a customized NGS panel targeting 156 or 733 cancer-related genes (the full list was provided in Supplementary Table 1). Based on the annotation of UCSC Genome RepeatMasker, we filtered out repetitive elements from intronic baits [16]. The xGen® Hybridization and Wash Kit (IDT) was used for hybridization enrichment following the manufacturer’s instructions. Briefly, we added 500 ng indexed DNA libraries to each well containing Blocker Master Mix and pooled to obtain a total amount of 2 μg of DNA. Then, the pooled DNA sample was mixed with human cot DNA and xGen Universal Blockers-TS Mix, and the mixture was dried using a SpeedVac system. The Hybridization Master Mix was added to the samples and denatured in a thermal cycler for 10min at 95°C, before being mixed and hybridized with 4 μl of probes at 65°C overnight. The target regions were captured following the manufacturer’s instructions. The Qubit 3.0 fluorometer (Thermo Fisher Scientific) and LabChip GX Touch HT Analyzer (PerkinElmer) were used to evaluate the final library’s concentration and fragment size distribution, respectively.

### DNA sequencing, data processing, and variant calling

The qualified DNA libraries were captured and loaded onto an Illumina NovaSeq 6000 platform using 100 bp paired-end read sequencing. Sequencing Raw data with paired samples (FFPE and its normal control) were aligned to the reference human genome hg19 using the Burrows-Wheeler Aligner (v0.7.12) [17]. PCR duplicate reads were identified with Picard tools (v1.130), and non-uniquely mapping reads were removed using SAMtools (v1.1.19). Variant calling was only performed in the targeted regions. Signatures of the somatic single nucleotide variants (SNVs) were investigated using an in-house developed R package to execute a variant detection model based on standard binomial distributions. Local realignment around insertions/deletions (indels) was performed to avoid misalignments. Variants were further filtered based on their unique supporting read depth, base quality, and strand bias as previously described [18].

All variants were then further filtered to ensure sensitivity and specificity at an allele frequency (AF) of ≥1% using an automated false positive filtering pipeline. Filtered single-nucleotide polymorphism (SNPs) and indels were annotated and categorized by ANNOVAR software against the following databases: dbSNP (v138), 1000Genome, and ESP6500 (population frequency > 0.015). Only frameshift, non-frameshift, stop gain and missense, and indel mutations were included. Gene rearrangements and copy number variations (CNVs) in 156 or 733 target genes were detected as described previously (Supplementary Table 1) [18]. Tumor mutation burden (TMB) was estimated as the average number of synonymous and non-synonymous somatic mutations per megabase pair (Mbp) in the genome, including SNVs and indels in examined coding regions and excluding known cancer driver mutations. All SNVs and indels in the coding region of targeted genes, including silent, nonsense, missense, splice site, stop loss, stop gain, frameshift, and in-frame mutations, were kept.

### Homologous recombination deficiency analysis

For estimating genomic scar, we developed the 3DMed-HRD algorithm [19–21] based on over 10,000 single-nucleotide polymorphisms distributed across the human genome, which were also included in 156- and 733-gene NGS assays (detailed gene lists are shown in Supplementary Table 1). HRR pathway genes were designated according to previous studies (Supplementary Table 1). All mutations in this study referred to deleterious or suspected deleterious ones.

LOH score referred to the number of intermediate-size LOH regions (shorter than the whole chromosome and longer than 15 Mb) [22]. TAI score referred to the number of subchromosomal regions with allelic imbalance extending to the telomere without crossing the centromere [23]. LST score referred to the number of chromosomal breaks between adjacent regions of longer than 10 Mb [24]. HRD score is the sum of LOH score, TAI score, and LST score, adjusted by tumor ploidy and purity.

### Cut-off and endpoints definition

Similar to MyChoice CDx, 3DMed-HRD algorithm was developed using a Chinese breast cancer and ovarian cancer cohort as training set (Supplementary Figure 1). There is currently a lack of optimal HRD cut-off value, so the rank-sum test for the quantitative HRD scores, the threshold of 21 (median HRD score in our prostate cancer cohort), and 30 (based on 95% sensitivity for BRCA-deficiency detection in the training ovarian and breast cancer cohort) were all analyzed in this study; some results were displayed as supplementary data.

CFS (CRPC-free survival), MFS (metastasis-free survival), and OS (overall survival) were defined as the time from prostate cancer diagnosis to castration-resistant prostate cancer (CRPC), metastasis disease (only for M0 patients), and death, respectively.

### Limit of detection (LoD) for analytical sensitivity, repeatability, and reproducibility

HCC1143 cell line (with marked genetic instability) was purchased from ATCC and used to simulate tumor for validation [24]. We diluted the extracted DNA from these cells to purity gradient as 50%, 40%, 30%, 20%, 10%, 5%, and 0, respectively. Three replicates of each of the various DNA mixes were run and analyzed to determine the LoD of these HRD scores.

We use two clinical samples (59166S01 and 60016S01) to assess the accuracy of our HRD score detection. Two groups were repeated for each sample. Each reaction was repeated in triplicate and all experiments were repeated at least twice to confirm inter- and intra-batch repeatability and reproducibility.

### Statistics

Data analyses were performed using R (version 4.0.5, 2021). Kruskal-Wallis rank-sum tests (HRD scores were coded as numeric variables) and chi-square tests (HRD scores were coded as categorical variables) were used for continuous and discrete variables, respectively. All *P* values were two-sided. Kaplan-Meier plot and Cox proportional hazards regression were used for survival analyses.

## Results

### Limit of detection (LoD), repeatability, and reproducibility

When tumor purity ≥ 20%, all pools of HRD-positive status could be called out in HCC1143 cell line series dilution with 500X mean coverage. Thus, we set the LOD of HRD-positive status as tumor purity 20% (Supplementary Figure 2. A).

Two clinical samples with different HRD scores were repeated for the cancer panel assay. In addition, the average coefficient of variation (CV) of the two samples was 2.44%. For the sample set analyzed, the targeted cancer panel meets or exceeds the criteria set forth in the validation plan (Supplementary Figure 2. B).

### Patient characteristics and overall HRD score distribution

**Fig. 1:**
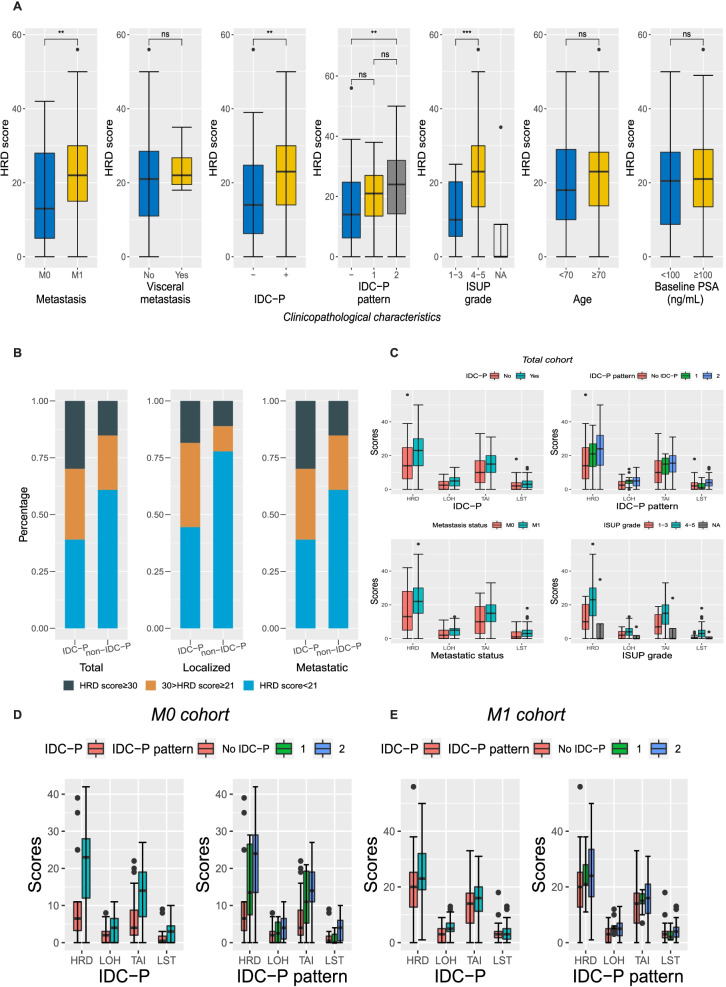
The association of HRD scores with IDC-P and other clinicopathological characteristics. **A** Boxplots showing HRD score distribution in various clinicopathological characteristic settings (metastatic status, visceral metastasis, IDC-P, IDC-P pattern, ISUP grade, age, baseline PSA). **B** Stacking histogram showing that IDC-P and non-IDC-P tumors display different proportions of HRD scores (divided by threshold (21, 30)) in the total, localized, and metastatic cohorts, respectively. **C** Boxplots showing the distribution of HRD score, as well as LOH score, LST score, and TAI score, in IDC-P (negative/positive), IDC-P pattern (negative/1/2), metastatic status (negative/positive), and ISUP grade (1–3/4–5) groups in the total cohorts. **D**, **E** Boxplots showing the distribution of HRD score, as well as LOH score, LST score, and TAI score, in IDC-P (negative/positive), IDC-P pattern (negative/1/2), in the M0 and M1 cohorts, respectively. M0, localized; M1, de novo metastatic; IDC-P, intraductal carcinoma of the prostate, PSA, prostate-specific antigen; ISUP, International Society of Urological Pathology; HRD, homologous recombination deficiency

The median HRD score of this cohort is 21.0 (IQR: 11.0, 29.0), with 52.8% (65/123) patients showing HRD score≥21, and 24.3% (30/123) patients showing HRD score≥30. There are a total of nine patients displaying HRD scores as zeros, and the percentage in non-IDCP was more than three times that in the IDC-P group, being 3/77 (3.9%) in IDC-P and 6/46 (13.0%) in non-IDC-P (*P*=0.059), respectively. Clinicopathological features including M1 (77/123, 62.6%) (median: 22.0 vs. 13.0, *P*=0.008), IDC-P (77/123, 62.6%) (median: 23.0 vs. 14.0, P=0.002), and International Society of Urological Pathology (ISUP) grades≥4 (103/123, 83.7%) (median: 23.0 vs. 10.0, *P*=0.001) were correlated to high HRD scores (Fig. 1A, Table 1).

**Table 1:** The association of prostate cancer clinicopathological features and HRD scores in total, localized, and metastatic cohorts

		HRD as continuous variable			HRD as dichotomous variable			
		Mean (std)	Median (IQR)	***P*** value^**1**^	HRD score≥21	***P*** value^**2**^	HRD score≥30	***P*** Value^**3**^
**Total cohort**	**Total (** ***n*** **=123)**	20.81 (12.83)	21.00 (11.00, 29.00)		65 (52.9%)		30 (24.3%)	
	**IDC-P**							
	**IDC-P (+),** ***N*** **=77**	23.00 (12.11)	23.00 (14.00, 30.00)	0.002	47 (61.0%)	0.019	23 (29.8%)	0.107
	**IDC-P (-),** ***N*** **=46**	16.52 (12.98)	14.00 (5.75, 25.00)		18 (39.1%)		7 (15.2%)	
	**IDC-P pattern**							
	**Pattern 1,** ***N*** **=19**	20.21 (9.60)	21.00 (13.00, 28.00)	0.005*	11 (57.9%)	0.059*	3 (15.7%)	0.048*
	**Pattern 2,** ***N*** **=58**	24.41 (12.73)	24.00 (14.00, 32.75)		36 (62.1%)		20 (34.4%)	
	**Metastasis**							
	**Localized,** ***N*** **=45**	16.33 (12.92)	13.00 (4.50, 28.00)	0.008	19 (42.2%)	0.073	7 (15.5%)	0.130
	**mPCa,** ***N*** **=78**	23.40 (12.12)	22.00 (14.75, 30.50)		46 (59.0%)		23 (29.4%)	
	**ISUP grade**							
	**Grades 1–3,** ***N*** **=16**	11.50 (8.73)	10.00 (5.50, 20.25)	0.001	4 (25.0%)	0.013	0	0.015
	**Grades 4–5,** ***N*** **=103**	22.73 (12.40)	23.00 (13.50, 30.00)		60 (58.3%)		29 (28.2%)	
	**NA,** ***N*** **=4**	8.75 (17.5)	0		1 (25%)		1 (25%)	
**Localized cohort**	**IDC-P**							
	**IDC-P (+),** ***N*** **=27**	20.04 (12.34)	23.00 (11.00, 28.00)	0.017	15 (55.6%)	0.027	5 (18.5%)	0.801
	**IDC-P (-),** ***N*** **=18**	10.78 (12.03)	6.50 (2.50, 14.50)		4 (22.2%)		2 (11.1%)	
	**IDC-P pattern**							
	**Pattern 1,** ***N*** **=8**	15.50 (10.98)	13.50 (6.50, 27.50)	0.038*	3 (37.5%)	0.040*	0	0.181*
	**Pattern 2,** ***N*** **=19**	21.95 (12.65)	24.00 (13.00, 30.00)		12 (63.2%)		5 (26.3%)	
**Metastatic cohort**	**IDC-P**							
	**IDC-P (+),** ***N*** **=50**	25.18 (11.71)	23.00 (18.75, 32.00)	0.065	32 (64.0%)	0.228	18 (36.0%)	0.154
	**IDC-P (-),** ***N*** **=28**	20.21 (12.40)	20.00 (12.25, 25.75)		14 (50.0%)		5 (17.8%)	
	**IDC-P pattern**							
	**Pattern 1,** ***N*** **=11**	21.83 (11.70)	20.50 (13.75, 30.00)	0.173*	8 (72.7%)	0.387*	3 (27.3%)	0.187*
	**Pattern 2,** ***N*** **=39**	27.06 (11.47)	25.50 (20.00, 35.75)		24 (61.6%)		15 (38.5%)	

*P* value^1^: Comparing HRD score as continuous variables (Rank-sum test); *P* value^2^: comparing HRD score ≥21 vs. <21 (chi-square test); *P* value^3^: comparing HRD score ≥30 vs. <30 (chi-square test)

*HRD* homologous recombination deficiency, *IDC-P* intraductal carcinoma of the prostate, *mPCa* metastatic prostate cancer, *std* standard deviation, *IQR* interquartile range

*IDC-P (-) vs. IDC-P pattern 1 vs. IDC-P pattern 2

### Tumors with IDC-P display higher HRD scores

Approximately three-fifths of patients had IDC-P (77/123, 62.6%). Patients with IDC-P pattern 2 (58/123, 47.2%) were about three times as many as those with IDC-P pattern 1 (19/123, 15.4%), consistent with the previously reported natural distribution of IDC-P subtypes [9, 10]. Baseline characteristics were comparable between the IDC-P and the non-IDC-P groups (Table 2).

**Table 2:** Baseline characteristics of the total cohort and cases with or without IDC-P

		All patients (***N***=123)	Without IDC-P (***N***=46)	With IDC-P (***N***=77)	***P*** value
**HRD score (median [IQR])**		21.0 [11.0, 28.5]	14.0 [6.2, 24.8]	23.0 [14.0, 30.0]	0.002
**HRD score (%)**	<21	58 (47.15)	28 (60.87)	30 (38.96)	0.019
	≥21	65 (52.85)	18 (39.13)	47 (61.04)	
**Metastasis (%)**	No	45 (36.59)	18 (39.13)	27 (35.06)	0.795
	Yes	78 (63.41)	28 (60.87)	50 (64.94)	
**Visceral metastasis (%)**	No	119 (96.75)	44 (95.65)	75 (97.40)	0.997
	Yes	4 (3.25)	2 (4.35)	2 (2.60)	
**IDCP pattern (%)**	0	46 (37.40)	46 (100.00)	0 (0.00)	<0.001
	1	19 (15.45)	0 (0.00)	19 (24.68)	
	2	58 (47.15)	0 (0.00)	58 (75.32)	
**ISUP/WHO (%)**	1-3	20 (16.26)	10 (21.74)	10 (12.99)	0.308
	4-5	103 (83.74)	36 (78.26)	67 (87.01)	
**Age (median [IQR])**		69.0 [64.0, 73.5]	69.5 [67.0, 75.0]	68.0 [62.0, 73.0]	0.235
**Age (%)**	<70	67 (54.47)	23 (50.00)	44 (57.14)	0.56
	≥70	56 (45.53)	23 (50.00)	33 (42.86)	
**PSA (median [IQR])**		67.8 [19.6, 100.1]	75.1 [28.7, 100.1]	60.0 [15.3, 100.1]	0.187
**Baseline PSA (ng/mL, %)**	<100	72 (58.54)	26 (56.52)	46 (59.74)	0.872
	≥100	51 (41.46)	20 (43.48)	31 (40.26)	

*HRD* homologous recombination deficiency, *IDC-P* intraductal carcinoma of the prostate, *ISUP* International Society of Urological Pathology, *PSA* prostate-specific antigen, *IQR* interquartile range

Patients with and without IDC-P did not differ in DDR pathway (44/77, 57.1% vs. 19/46, 41.3%, *P*=0.130), HRR pathway (25/77, 32.5% vs. 10/46, 21.7%, *P*=0.285), and *BRCA* (11/77, 14.3% vs. 6/46, 13.0%, *P*=1.000) mutation rate. However, tumors with IDC-P showed significantly higher median HRD scores than AC samples (23.0 vs. 14.0, *P*=0.002) (Table 1). IDC-P and non-IDC-P tumors contained 72.3% (47/65) and 27.7% (18/65) of cases with HRD≥21 (*P*=0.019), respectively (Fig. 1B). Tumors with IDC-P showed higher HRD-LOH score (median: 5.0 vs. 2.5, *P*=0.001) and HRD-LST score (median: 15.0 vs. 10.0; *P*=0.009), while HRD-TAI score showed a borderline significance (median: 3.0 vs. 2.0, *P*=0.076). The median HRD, HRD-LOH, and HRD-LST scores were numerically ladder-shaped among IDC-P (-), IDC-P pattern 1, and IDC-P pattern 2 groups, except for the case of HRD-TAI scores (Fig. 1C, Supplementary Table 2).

IDC-P was associated with higher HRD scores in M0 patients (median: 23.0 vs. 6.5, *P*=0.017), while it was just seemingly correlated with higher HRD scores in M1 patients (median: 23.0 vs. 20.0, *P*=0.065). The numerical ladder shape for HRD scores in IDC-P subtype groups is basically maintained in the M0 patients (Fig. 1D, Table 1). Nevertheless, in the M1 group, the HRD scores were similar between patients without IDC-P and with IDC-P pattern 1, while those with IDC-P pattern 2 were still a little higher (Fig. 1E, Table 1). HRD-TAI score was not different between IDC-P and non-IDCP tumors in both M0 and M1 groups. HRD-LST score was correlated with IDC-P in the M0 cohort, while HRD-LOH score was correlated with IDC-P in the M1 cohort, respectively (Fig. 1D, E, Table 1).

### The association of HRD scores with genomic mutations

**Fig. 2:**
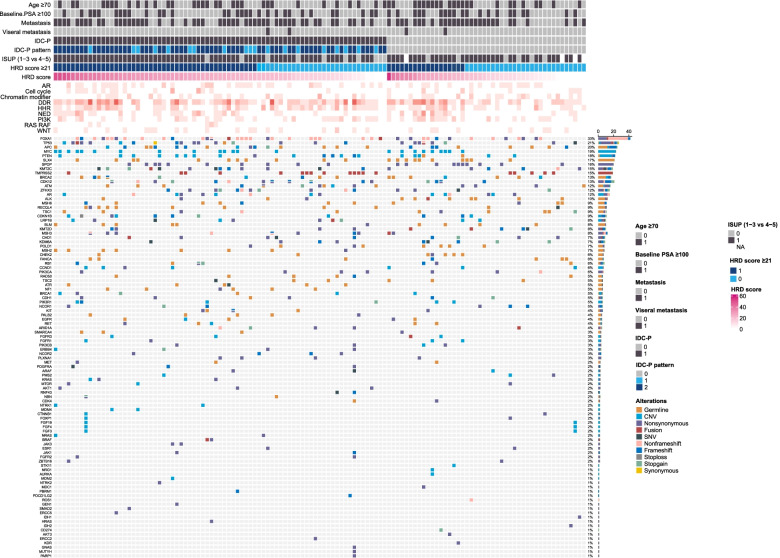
Heatmap showing the genomic alterations in this cohort grouped by clinicopathological characteristics (age, baseline PSA, metastatic status, visceral metastasis, IDC-P, IDC-P pattern, ISUP grade) and HRD score. M0, localized; M1, de novo metastatic; IDC-P, intraductal carcinoma of the prostate; PSA, prostate-specific antigen; ISUP, International Society of Urological Pathology; HRD, homologous recombination deficiency

As anticipated, mutations of *BRCA*, and HRR pathway genes were associated with higher HRD scores in the whole cohort [*BRCA*-m (*n*=17) vs. *BRCA*-wt (*n*=106): median: 32.0 vs. 20.5, *P*=0.019; HRR-m (*n*=35) vs. HRR-wt (*n*=88): median: 24.0 vs. 20.0, *P*=0.045]. When removing *BRCA* mutations from HRR pathway, other HRR pathway gene mutations were not correlated with HRD scores, suggesting *BRCA* mutations were still the main contributors of HRD score within HRR pathway (median: 20.7 vs. 21.2, *P*=0.788). Genomic alterations in this cohort grouped by IDC-P presence are shown in Fig. 2. Also, within the *BRCA*-wt group (*n*=106), patients with IDC-P (*n*=66) showed significantly higher HRD score [median: 23.0 vs 13.0, *P*=0.002; HRD score≥21: 39/66 (59.1%) vs. 14/40 (35.0%), *P*=0.016].

**Fig. 3:**
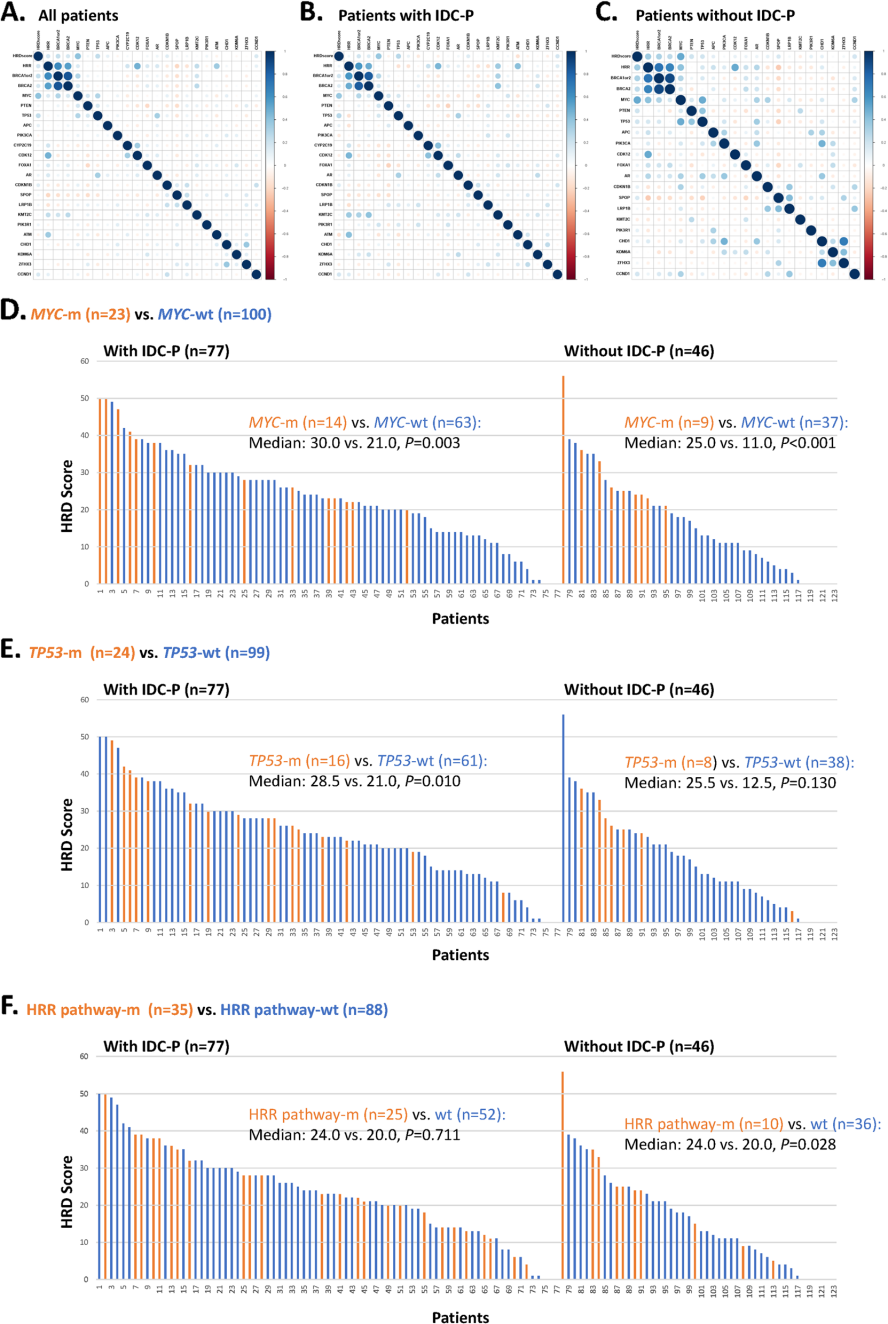
**A**–**C** The correlation analyses of the top mutated genes (genes mutated in ≥5 cases) and HRD scores in the total, IDC-P, and non-IDC-P cohorts. **D**–**F** Histograms showing the HRD score of each patient in the IDC-P and non-IDC-P cohorts. Different colors represent the mutation status of *MYC* (**D**), *TP53* (**E**), and HRR pathway genes (**F**): yellow=mutated, blue=wild type. IDC-P, intraductal carcinoma of the prostate; HRR, homologous recombination repair

Besides *BRCA*, the correlation analyses of the top mutated genes (genes mutated in ≥5 cases) and HRD scores also relate *MYC* and *TP53* mutations to higher HRD scores [*MYC*-m (*n*=23) vs. *MYC*-wt (*n*=100): median: 26.0 vs. 18.5, *P*<0.001; *TP53*-m (*n*=24) vs. *TP53*-wt (*n*=99): median: 28.0 vs. 20.0, *P*=0.002] (Fig. 3A–C). Unlike *MYC*, which was an indicator of elevated HRD scores in both IDC-P (*P*=0.003) and non-IDC-P (*P*<0.001) cohorts, *TP53* (Fig. 3D, E) mutations indicated high HRD scores in IDC-P (*P*=0.010) but not the non-IDC-P (*P*=0.130) patients. In contrast, raised HRD scores in non-IDC-P patients were predominantly driven by HRR pathway mutations (*P*=0.028) (Fig. 3F).

### Prognostic significance of HRD scores

At the end of follow-up, ten and eight patients in the M0 cohort progressed to metastatic disease and CRPC, respectively. About half of the M1 patients (39/77, 50.6%) developed into CRPC. Fifteen patients died during the follow-up, with 12/15 (80.0%) of them harboring an HRD score higher than 21. We analyzed the impact of HRD scores on patients’ CFS and OS. Since M0 and M1 patients have disparate prognoses, we further performed the survival analyses separately. The deceased patients included 14 M1 patients and only one M0 patient; thus, MFS was used as the second endpoint instead of OS in the M0 group.

**Fig. 4:**
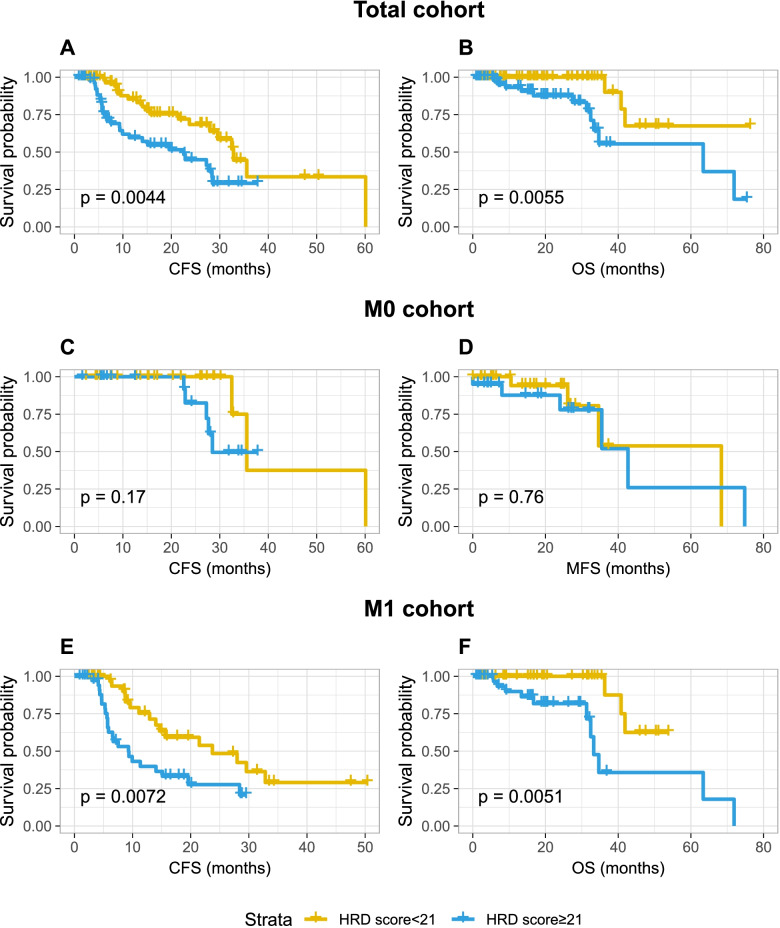
Prognostic value of HRD score. **A**, **B** HRD score in predicting CFS and OS time in the total cohort. **C**, **D** HRD score in predicting CFS and OS time in the M0 cohort. **E**, **F** HRD score in predicting CFS and OS time in the M1 cohort. M0, localized; M1, de novo metastatic; IDC-P, intraductal carcinoma of the prostate; CFS, CRPC-free survival; MFS, metastasis-free survival; OS, overall survival; HRD, homologous recombination deficiency

In the total cohort, high HRD scores (≥21) were correlated with shorter CFS (22.6 vs. 32.8 months, *P*=0.004) and OS (63.4 months vs. not reached, *P*=0.005) (Fig. 4A, B). Regarding the M0 group, there were still drastic differences in median survival time, though they were not statistically significant, probably due to the small sample sizes (HRD≥21 vs. <21: CFS: 28.5 vs. 35.6 months, MFS: 42.7 vs. 68.4 months) (Fig. 4C, D). Similar to the total cohort, adverse prognostic significance was also found in M1 group (HRD≥21 vs. <21: MFS: 9.3 vs. 23.7 months, *P*=0.007; OS: 33.3 months vs. not reached, *P*=0.005) (Fig. 4E, F).

## Discussion

This study firstly reported that tumors with IDC-P components showed significantly higher HRD scores than AC. By using a cohort containing both localized and metastatic patients, we found that M1 patients were more likely to show higher HRD scores than M0 patients. We identified that the mutations of genes other than the HRR pathway (*MYC* and *TP53*) also indicate high HRD scores in PCa. While HRR pathway gene alterations mainly drove the high HRD score phenotype in tumors without IDC-P, *TP53* mutation exclusively indicated high HRD scores in IDC-P tumors. This discrepancy has not been observed before and could have important molecular implications. Also, we found that tumors with HRD scores showed worse survival in aggressive prostate cancers.

Since the mutational signature-based HRDetect algorithm identified that over 30% of the HRD-high score tumors showed no *BRCA* mutations and mutations in other homologous recombination genes [6], HRD is probably associated with gene aberrations other than the canonical HRR pathway. One of them is *MYC*, which has been noticed to control DNA double-strand break repair in several tumors, and the combination of *MYC* blockade with PARP inhibitors can be independent of *BRCA* status [25–28]. Previous reports have shown the association of *TP53* mutations with chromosomal instability, copy number change, and HRD scores [29, 30–32]. As expected, our data showed that the HRD score in tumors without IDC-P was mainly associated with HRR pathway gene mutations. In contrast, high HRD scores in tumors with IDC-P were possibly driven by *TP53* alteration instead. This finding further highlights the possible heterogeneity underlying genome maintenance mechanism between different PCa pathological subtypes. Therefore, IDC-P is likely to differ from AC regarding driver mutations for chromosomal instability, e.g., loss of cell-cycle checkpoint mechanisms [32]. This finding requires more in-depth functional research to unmask whether different mechanisms exist in IDC-P in mediating HRD. Collectively, the HRD score is indispensable in understanding tumors’ HRD status apart from the *BRCA* mutation test.

IDC-P is considered frequently co-existed with a constellation of negative indexes, including genomic instability [14, 15, 33]. Our results work in concert with these observations to reflect a high global genomic instability in IDC-P through HRD score calculation. Therefore, patients with IDC-P should be recommended to perform HRD score tests. Also, all samples used in this study were collected at the time of their initial diagnosis of PCa, precluding the treatment interference. The results that M1 tumors showing higher HRD scores indicate that the genomic scars may accumulate during the natural disease course of PCa. The already existing genomic scars are unlikely to disappear even with the restoration of the HR function. Thus, high-risk patients are highly recommended to perform HRD tests in the earlier phase of their disease, and preferably require repeated tests when the disease progresses, to ensure the optimal therapeutic strategy is used at the best possible time.

Furthermore, we discovered that HRD scores have significant prognostic value in aggressive prostate cancers. This is very informative to clinicians since conventional prognostic markers such as the Gleason score can be of little use in stratifying highly aggressive diseases. With the help of HRD score results, clinicians are able to consider more upfront therapies and pay extra attention to the efficacy of PARP inhibitors in patients with high HRD scores.

This study has a few limitations. First, because PARPi has not been widely used in the Chinese market for PCa treatment due to socioeconomic reasons, we lack the corresponding data to confirm the use of HRD score in predicting the responses for PARPi. Also, as PCa exhibits lower HRD scores [5], simply using this threshold for breast cancer and ovarian cancer may not be appropriate. So we used the Kruskal-Wallis rank-sum tests and two cut-off points (the median value of this cohort and the threshold for breast cancer and ovarian cancer) in that there is currently no acknowledged threshold value for HRD score in PCa; a similar approach is shared by Lotan et al. in their recent article about HRD in PCa [31]. Furthermore, we used a panel-based approach to detect genomic mutations; thus, some alternative mechanisms for increased HRD scores could be neglected. Last, we could not compare our test results with Myriad myChoice CDx since it is not available in our region yet. We hope in the future we can compare the consistency between our algorithm and theirs.

## Conclusions

De novo metastatic PCa patients and patients with IDC-P or high ISUP grades harbored higher HRD scores. HRD status in IDC-P patients can derive from abnormities other than the HRR pathway. High-risk prostate cancer patients are recommended to perform the HRD score test since the results can be a useful predictive and prognostic marker in aggressive prostate cancers.

## Supplementary Information

**Additional file 1: Supplementary Table 1**. Sheet 1: 733-gene Next Generation Sequencing (NGS) panel gene list. Sheet 2: 156-gene NGS panel gene list. Sheet 3: Annotation of homologous recombination repair (HRR) genes used in this study.

**Additional file 2: Supplementary Figure 1**. HRD, HRD-LOH, HRD-TAI, HRD-LST scores of BRCA1/2-intact and BRCA1/2-deficient samples in the training breast and/or ovarian cancer set. HRD: homologous recombination deficiency.

**Additional file 3: Supplementary Figure 2**. A. scatter diagram showing the detected HRD scores for different tumor purity gradient (50%, 40%, 30%, 20%, 10%, 5%, 0); red dotted line: tumor purity = 20%; blue dotted line: the cut-off value 21. B. red dotted line: average HRD score of the clinical samples; blue dashed line: the cut-off value 21; points: inter-batch.

**Additional file 4: Supplementary Table 2**. The association of prostate cancer clinicopathological features and LOH, TAI and LST in total, localized and metastatic cohorts.

## Data Availability

Some data in this study is available upon reasonable request through the corresponding author.

## Abbreviations

AC: Acinar adenocarcinoma
ADT: Androgen deprivation therapy
DDR: DNA damage repair
HRD: Homologous recombination deficiency
HRR: Homologous recombination repair
IDC-P: Intraductal carcinoma of the prostate
M0: Localized
M1: Metastatic
MAB: Maximal androgen blockade
PARPi: Poly ADP-ribose polymerase inhibitor
PCa: Prostate cancer
wt: Wild type
