# Supplementary figures and images for: Homologous recombination deficiency (HRD) score in aggressive prostatic adenocarcinoma with or without intraductal carcinoma of the prostate (IDC-P)
Source: BMC Med. 2022 Jul 22;20:237. doi: 10.1186/s12916-022-02430-0 (PMC9306093; doi:10.1186/s12916-022-02430-0)

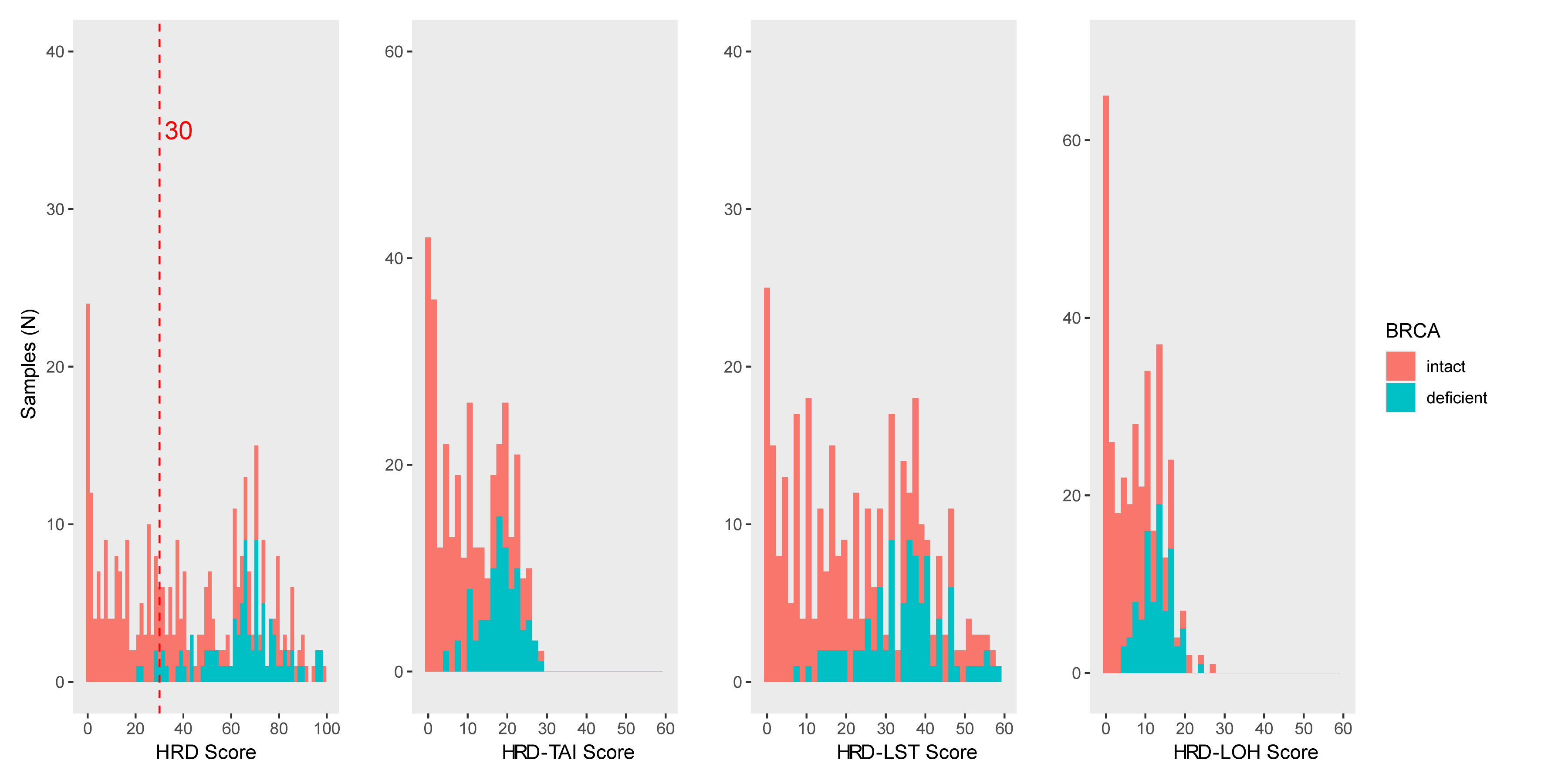

Supplement: Supplementary file 2 — Additional file 2: Supplementary Figure 1. HRD, HRD-LOH, HRD-TAI, HRD-LST scores of BRCA1/2-intact and BRCA1/2-deficient samples in the training breast and/or ovarian cancer set. HRD: homologous recombination deficiency. [file 12916_2022_2430_MOESM2_ESM.jpg]

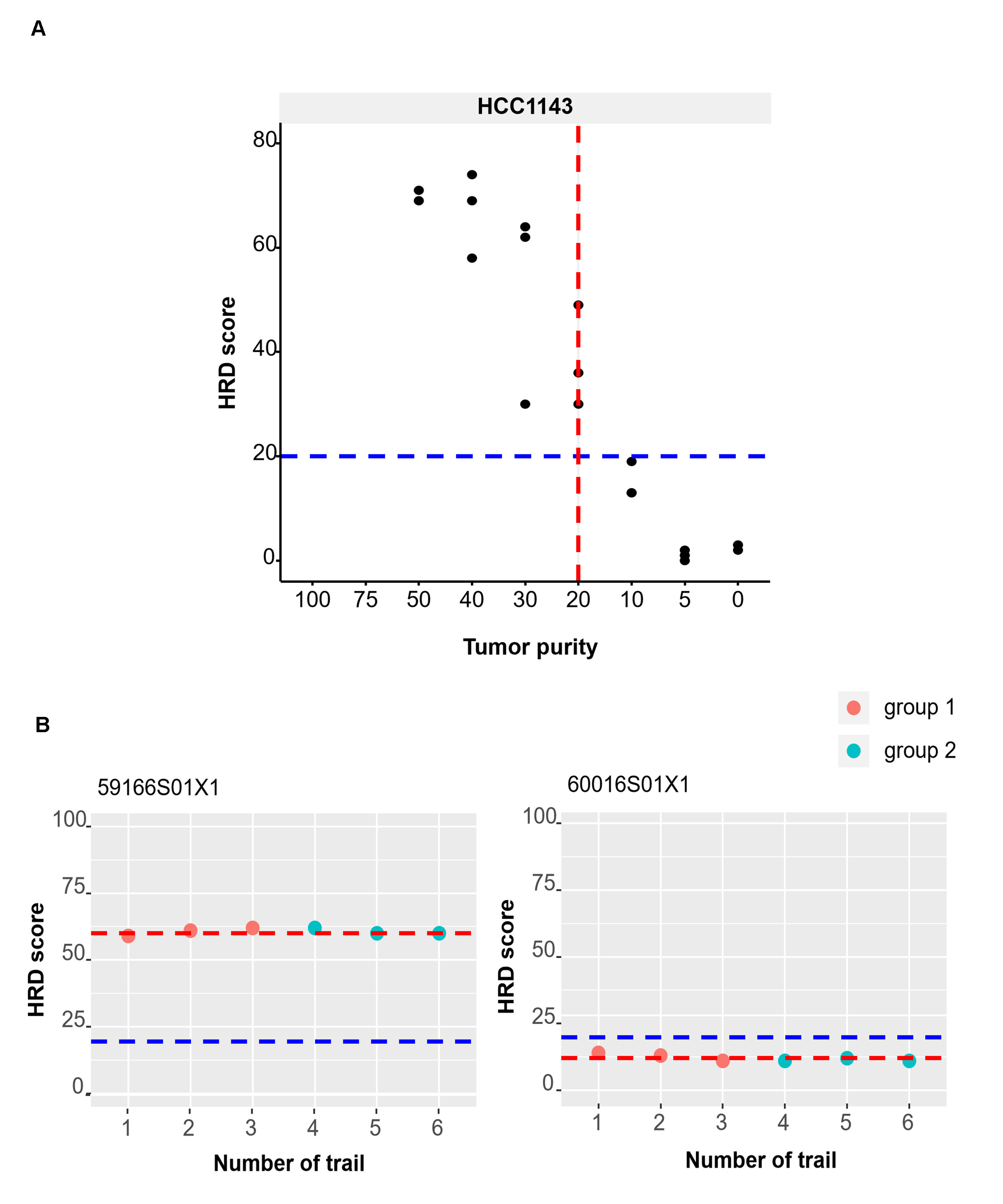

Supplement: Supplementary file 3 — Additional file 3: Supplementary Figure 2. A. scatter diagram showing the detected HRD scores for different tumor purity gradient (50%, 40%, 30%, 20%, 10%, 5%, 0); red dotted line: tumor purity = 20%; blue dotted line: the cut-off value 21. B. red dotted line: average HRD score of the clinical samples; blue dashed line: the cut-off value 21; points: inter-batch. [file 12916_2022_2430_MOESM3_ESM.jpg]
