# Supplementary material for: Homologous recombination deficiency (HRD) score in aggressive prostatic adenocarcinoma with or without intraductal carcinoma of the prostate (IDC-P)
Source: BMC Med. 2022 Jul 22;20:237. doi: 10.1186/s12916-022-02430-0 (PMC9306093; doi:10.1186/s12916-022-02430-0)
Supplement: Supplementary file 4 — Additional file 4: Supplementary Table 2. The association of prostate cancer clinicopathological features and LOH, TAI and LST in total, localized and metastatic cohorts. [file 12916_2022_2430_MOESM4_ESM.docx]

**Table S2. The association of prostate cancer clinicopathological features and LOH, TAI and LST in total, localized and metastatic cohorts.**

|  |  | **LOH** | | |  | **TAI** | | |  | **LST** | | |
| --- | --- | --- | --- | --- | --- | --- | --- | --- | --- | --- | --- | --- |
|  |  | **Mean (std)** | **Median (IQR)** | ***P* value^1^** |  | **Mean (std)** | **Median (IQR)** | ***P***  **value^1^** |  | **Mean (std)** | **Median (IQR)** | ***P***  **value^1^** |
| **Total cohort** | **Total, N=123** | 3.96 (3.09) | 4.00 (1.00, 6.00) |  |  | 3.37 (3.47) | 3.00 (1.00, 4.00) |  |  | 13.49 (8.00) | 14.00 (7.00, 19.00) |  |
|  | **IDC-P** |  |  |  |  |  |  |  |  |  |  |  |
|  | **IDC-P (+), N=77** | 4.70 (3.15) | 5.00 (2.00, 7.00) | 0.001 |  | 3.71 (3.48) | 3.00 (1.00, 5.00) | 0.076 |  | 14.96 (7.36) | 15.00 (10.00, 20.00) | 0.009 |
|  | **IDC-P (-), N=46** | 2.72 (2.56) | 2.50 (0.00, 5.00) |  |  | 2.78 (3.43) | 2.00 (0.00, 4.00) |  |  | 11.02 (8.50) | 10.00 (3.75, 17.00) |  |
|  | **IDC-P pattern** |  |  | 0.003* |  |  |  | 0.012* |  |  |  | 0.025* |
|  | **Pattern 1, N=19** | 4.68 (3.15) | 5.00 (3.00, 6.00) |  |  | 2.05 (2.09) | 1.00 (0.00, 4.00) |  |  | 13.47 (6.11) | 15.00 (8.00, 19.00) |  |
|  | **Pattern 2, N=58** | 4.71 (3.18) | 5.00 (2.00, 7.00) |  |  | 4.26 (3.67) | 4.26 (3.673) |  |  | 15.45 (7.71) | 15.50 (10.00, 20.25) |  |
|  | **Metastasis** |  |  |  |  |  |  |  |  |  |  |  |
|  | **Localized, N=45** | 3.09 (3.06) | 2.00 (0.00, 5.50) | 0.018 |  | 2.47 (2.97) | 1.00 (0.00, 4.00) | 0.009 |  | 10.78 (8.32) | 10.00 (3.00, 19.00) | 0.008 |
|  | **mPCa, N=78** | 4.46 (3.01) | 5.00 (2.00, 6.00) |  |  | 3.88 (3.65) | 3.00 (1.00, 5.00) |  |  | 15.05 (7.43) | 15.00 (9.75, 20.00) |  |
|  | **ISUP grade** |  |  |  |  |  |  |  |  |  |  |  |
|  | **Grade 1-3, N=16** | 2.50 (2.28) | 2.00 (0.00, 4.25) | 0.031 |  | 0.88 (1.20) | 0.50 (0.00, 1.00) | 0.001 |  | 8.12 (6.51) | 7.00 (2.75, 14.25) | 0.002 |
|  | **Grade 4-5, N=103** | 4.27 (3.11) | 4.00 (2.00, 6.00) |  |  | 3.84 (3.56) | 3.00 (1.00, 5.00) |  |  | 14.61 (7.63) | 15.00 (8.50, 20.00) |  |
| **Localized cohort** | **IDC-P** |  |  |  |  |  |  |  |  |  |  |  |
|  | **IDC-P (+), N=27** | 3.67 (3.22) | 4.00 (0.00, 7.00) | 0.137 |  | 3.07 (3.04) | 3.00 (0.00, 5.00) | 0.096 |  | 13.30 (8.10) | 14.00 (6.00, 19.00) | 0.024 |
|  | **IDC-P (-), N=18** | 2.22 (2.65) | 2.00 (0.00, 3.50) |  |  | 1.56 (2.68) | 0.50 (0.00, 2.00) |  |  | 7.00 (7.31) | 4.00 (1.75, 10.75) |  |
|  | **IDC-P pattern** |  |  | 0.269* |  |  |  | 0.022* |  |  |  | 0.066* |
|  | **Pattern 1, N=8** | 3.00 (3.12) | 2.50 (0.00, 6.50) |  |  | 1.13 (1.64) | 0.00 (0.00, 2.75) |  |  | 11.38 (8.21) | 11.00 (3.75, 19.75) |  |
|  | **Pattern 2, N=19** | 3.95 (3.31) | 4.00 (0.00, 7.00) |  |  | 3.89 (3.14) | 4.00 (0.00, 7.00) |  |  | 14.11 (8.14) | 14.00 (10.00, 19.00) |  |
| **Metastatic cohort** | **IDC-P** |  |  |  |  |  |  |  |  |  |  |  |
|  | **IDC-P (+), N=50** | 5.26 (3.00) | 5.00 (3.75, 7.00) | 0.001 |  | 4.06 (3.67) | 3.00 (1.00, 5.25) | 0.644 |  | 15.86 (6.85) | 16.00 (10.75, 20.00) | 0.196 |
|  | **IDC-P (-), N=28** | 3.04 (2.50) | 3.00 (0.00, 5.00) |  |  | 3.57 (3.66) | 3.00 (1.25, 4.00) |  |  | 13.61 (8.32) | 14.00 (7.00, 19.25) |  |
|  | **IDC-P pattern** |  |  | 0.005* |  |  |  | 0.423* |  |  |  | 0.397* |
|  | **Pattern 1, N=11** | 4.22 (1.73) | 4.50 (3.00, 5.25) |  |  | 3.33 (3.77) | 2.00 (1.00, 5.00) |  |  | 14.28 (7.84) | 14.00 (7.75, 21.25) |  |
|  | **Pattern 2, N=39** | 5.84 (3.41) | 6.00 (4.00, 8.00) |  |  | 4.47 (3.61) | 4.00 (2.00, 6.75) |  |  | 16.75 (6.18) | 17.00 (13.25, 20.00) |  |

**P value^1^: Comparing LOH, TAI and LST as continuous variables (Rank-sum test)**

*** IDC-P (-) vs. IDC-P pattern 1 vs. IDC-P pattern 2**

**LOH:** **loss of heterozygosity; TAI: telomeric allelic imbalance; LST: large-scale state transition; IDC-P: Intraductal carcinoma of the prostate; mPCa: metastatic prostate cancer; std: standard deviation; IQR: interquartile range**
